# Mitochondrial Dysfunction and Risk Factors for Noncommunicable Diseases: From Basic Concepts to Future Prospective

**DOI:** 10.3390/diseases12110277

**Published:** 2024-11-02

**Authors:** Ganna Nevoit, Gediminas Jarusevicius, Maksim Potyazhenko, Ozar Mintser, Inga Arune Bumblyte, Alfonsas Vainoras

**Affiliations:** 1 Laboratory of Population Studies, Cardiology Institute, Lithuanian University of Health Sciences, 44307 Kaunas, Lithuania; 2 Laboratory for Automatization of Cardiovascular Investigations, Cardiology Institute, Lithuanian University of Health Sciences, 44307 Kaunas, Lithuania; gediminas.jarusevicius@lsmu.lt; 3 Department of Internal Medicine and Emergency Medicine, Poltava State Medical University, 36011 Poltava, Ukraine; umsainua@ukr.net; 4 Department of Fundamental Disciplines and Informatics, Shupyk National Healthcare University of Ukraine, 04112 Kyiv, Ukraine; omintser@gmail.com; 5 Department of Nephrology, Lithuanian University of Health Sciences, 44307 Kaunas, Lithuania; ingaarune.bumblyte@lsmu.lt

**Keywords:** noncommunicable diseases, mitochondrial dysfunction, risk factors for noncommunicable diseases, diagnose mitochondrial dysfunction, disease prevention

## Abstract

Background/Objectives: Noncommunicable diseases (NCDs) are a very important medical problem. The key role of mitochondrial dysfunction (MD) in the occurrence and progression of NCDs has been proven. However, the etiology and pathogenesis of MD itself in many NCDs has not yet been clarified, which makes it one of the most serious medical problems in the modern world, according to many scientists. Methods: An extensive research in the literature was implemented in order to elucidate the role of MD and NCDs’ risk factors in the pathogenesis of NCDs. Results: The authors propose to take a broader look at the problem of the pathogenesis of NCDs. It is important to understand exactly how NCD risk factors lead to MD. The review is structured in such a way as to answer this question. Based on a systematic analysis of scientific data, a theoretical concept of modern views on the occurrence of MD under the influence of risk factors for the occurrence of NCDs is presented. This was done in order to update MD issues in clinical medicine. MD and NCDs progress throughout a patient’s life. Based on this, the review raised the question of the existence of an NCDs continuum. Conclusions: MD is a universal mechanism that causes organ dysfunction and comorbidity of NCDs. Prevention of MD involves diagnosing and eliminating the factors that cause it. Mitochondria are an important therapeutic target.

## 1. Introduction

Chronic noncommunicable diseases (NCDs) are a serious global socio-medical problem. The global NCD pandemic continues, despite the successes and advances of medicine in their treatment and prevention [1,2,3,4,5,6,7,8]. Therefore, all research aimed at solving the problem of NCDs is relevant. Over the past two decades, a large number of studies have been carried out that have shown the disruption of cellular energetics in NCDs and, accordingly, the important underlying role of mitochondrial dysfunction (MD) in this case. The great etiopathogenetic significance of MD in NCDs has been scientifically proven and is no longer in doubt [9,10,11]. However, the etiology and pathogenesis of MD itself in many diseases has not yet been clarified, which makes it one of the most serious medical problems in the modern world, according to many scientists. We fully agree with the opinion [11] that “the rapid advances in our knowledge of cellular metabolism coupled with the novel understanding at the molecular and genetic levels show tremendous promise to one day elucidate the mysteries of this ancient organelle in order to treat it therapeutically when needed”. MD is a definite therapeutic target because it is one of the key pathogenetic mechanisms of the onset and progression of NCDs. Understanding how and why MD occurs and progresses in NCDs is very significant for medicine. It is also important to understand why pathology occurs in certain organs in NCDs and what the significance of MD is in this case. Understanding these aspects may open up new therapeutic targets for the treatment and prevention of NCDs. This can help solve this important medical and social problem. For fundamental science and practical medicine, it is absolutely important to deepen our understanding of the systemic issues of the emergence and progression of NCDs and the role of MD therein.

Therefore, the aim of this theoretical study was to develop a systemic concept of the logic of the process of the occurrence and progression of NCDs, taking into account the role of MD as one of the universal pathogenetic mechanisms. For practical healthcare, an important issue is the development of a methodology for diagnosing MD as well as its prevention. Therefore, the review described methods for diagnosing MD and aspects of prevention which are based on the results of the concept of the causes of the onset and progression of MD in NCDs. For clinical medicine, it is important to understand exactly how NCDs’ risk factors lead to MD. Therefore, the review is structured in such a way as to answer this question.

It should be noted that NCDs are a group of chronic diseases, typically long-term, that result from a combination of genetic, physiological, environmental, and behavioral factors, as defined by the World Health Organization (WHO) [12]. The main types of NCDs are cardiovascular diseases (CVDs), cancers, chronic respiratory diseases, and diabetes [12]. These groups of NCDs cause 80% of human deaths worldwide. Understanding this commonality led science to the introduction of the term “NCDs” and the creation of general comprehensive programs for the prevention of NCDs [13,14,15,16,17,18]. The World Health Assembly extended the WHO Global action plan for the prevention and control of NCDs 2013–2020 to 2030 [12,19]. This is additional evidence of the importance of further scientific research in this area to solve the problem of NCDs throughout the world.

NCDs are associated with common risk factors [12]: unhealthy diet, physical inactivity, harmful use of alcohol, tobacco use, and environmental risk factors, which cause the entry of toxic substances into the human body.

These risk factors contribute to the development of four main metabolic disorders in the human body that increase the risk of NCDs: raised blood pressure, overweight/obesity, hyperglycemia (high blood glucose levels), and hyperlipidemia (high levels of fat in the blood).

It is well known that mitochondria are responsible for the implementation of the main metabolic processes in all cells of the human body. The role of MD has also been established in all NCDs [9,10,11]. However, the relationship between MD and risk factors for NCDs requires further description and scientific analysis. This is an important issue in the pathogenesis of NCDs. Shifting scientific attention to correcting mitochondrial function could change approaches to the treatment and prevention of NCDs and reduce the burden of NCDs for everyone in the world in the future. Therefore, the goal of this publication was to draw the attention of scientists to MD as one of the universal mechanisms of the pathogenesis of NCDs.

The review provides answers to questions such as: “When do MD and NCDs begin?”; “Due to what mechanisms do risk factors lead to the occurrence of MD and NCDs?”; “What is the role of MD in the occurrence and progression of NCDs?”; “Is the NCDs continuum valid?”; “How to prevent and diagnose MD in the clinical setting?”; “How to evaluate mitochondria and metabolic function in the clinical setting?”; and “What are the summary and future directions?”. 

## 2. When Do MD and NCDs Begin?

This is a very important question. In order to understand what causes lead to MD and NCDs, it is important to correctly find the “starting point” from which to begin scientific research. In the search for an answer to this question, attention was paid to CVDs as the largest group of NCDs.

It is indeed important to note that, for the global medical community, CVDs are the biggest medical problem among NCDs [4,5,20]. CVDs, including heart disease and stroke, are the world’s leading cause of death, claiming 18.6 million lives each year—33% of all global deaths. Of these deaths, 85% are due to ischemic heart diseases (e.g., heart attacks) and cerebrovascular diseases (e.g., strokes). Cases of CVDs nearly doubled from 271 million in 1990 to 550 million in 2019, with deaths rising from 12.1 million to 18.6 million across the same time period [21]. Therefore, the pathogenesis of CVDs is well studied in science, and it can be considered as a classic variant of the occurrence of pathology under the influence of NCDs risk factors. In order to answer the question “When do MD and NCDs occur?”, it is necessary to note the fact that CVDs have clinical manifestation solely against the background of metabolic changes in the body (for example, dyslipidemia, endothelial dysfunction, etc.). It has been scientifically established that the progression of CVDs is characterized by the emergence of a natural sequence of manifestations of pathology, which gradually change over time and complement existing preliminary disorders. This became the basis for the creation in 1991 of the Theory of the Cardiovascular Disease Continuum by V. Dzau and E. Braunwald [22]. The Cardiovascular Disease Continuum is a scientifically accepted description of the stages of development of CVDs [23,24,25,26,27,28]. An important proof is the fact that CVDs never occur suddenly and immediately in the human body. The clinical manifestation of CVDs is preceded by a period of formation of the so-called metabolic pattern (dyslipidemia, hypercholesterolemia, insulin resistance, etc.). This period of preclinical development was called by V. Dzau and E. Braunwald the stage of formation of risk factors of the cardiovascular continuum [29]. Thus, the stage of formation of risk factors is the entire period of time when the patient is still clinically healthy, but already leads an unhealthy lifestyle. Theoretically, this period can begin in childhood if the child receives an incorrect, unbalanced diet, leads a sedentary lifestyle, etc. This is a very logical argument for searching for the causes of MD and NCDs starting from childhood. In recent years, scientific research data have appeared on the occurrence of MD in children [29]. This confirms the idea that mitochondrial dysfunction, as a universal mechanism of NCDs, can begin to develop in childhood under the influence of risk factors.

Of course, scientific research in this direction must be continued, but this conclusion can be a starting point. Also, understanding that the problem of NCDs can arise as early as childhood can change approaches to its prevention.

## 3. Unhealthy Diet as an Etiological Factor in MD and NCDs

The state of energy metabolism at the microlevel of the structural organization (nanolevel and deeper: 10^−9^–10^−45^ cm) of the human body predetermines the transition of its cells from a state of health to a state of ill health [30,31,32,33]. These processes at the cellular level are associated with a disorder of the biogenesis and functioning of mitochondria, i.e., with mitochondrial dysfunction [9,10,11,34]. Inadequate, irrational nutrition with constant overeating is one of the main causes of NCDs’ risk factors [35,36,37,38,39,40]. But how exactly does poor nutrition cause the occurrence and development of MD?

In the pathogenesis of NCDs, the factor of malnutrition begins to act from childhood and continues to be of key importance throughout the entire period of the progression of NCDs. Under the influence of irrational nutrition with constant food overload, disorders of the processes of mitochondrial biogenesis occur, namely, the daily cycles of mitochondrial fission and fusion happen. Normally, daily cycles of mitochondrial fusion into a single mitochondrial network should occur in the cells of the human body. Mitochondria make up 10% of the human body weight [41]: in a person with a body weight of 70 kg, about 6–7 kg of body weight are mitochondria, that is, up to 10^15–17^ units. Mitochondria are semi-autonomous double-membrane organelles numbering 1–1500 units (except for mature red blood cells, which do not have mitochondria) with their own ring-shaped deoxyribonucleic acid (DNA). Depending on the amount of incoming metabolic substrates, mitochondria are found in different forms of mitochondrial dynamics: mitochondria merge into a mobile network of the mitochondrial reticulum when there is a deficiency of substrate (diet, starvation) and they go into a fragmented normal state when there is a sufficient supply of metabolic substrates [34,42]. This mitochondrial dynamic, and the constant movement of mitochondria in the cell with the ability to move between different cells in the body, brings mitochondria closer to individual single-celled organisms with their collective behavior and argues in favor of the theory of the bacterial origin of mitochondria. Therefore, a number of scientists propose to consider mitochondria as internal bacteria of the cells of the human body and call them “mitobiota”. At the same time, the variety of effects caused by mitochondria and symbiotic microorganisms became the basis for the concept of “mitobiota” and “microbiota” as two components of a single functional structure that regulates the homeostasis of the host organism through bioenergetic, epigenetic, metabolic, endocrine, immune, and neurohumoral connections [43,44,45,46,47,48].

The phenomenon of mitochondrial dynamics is very important for the health of the human body and the bioenergy supply of the cell because when there is a deficiency of food substrate, only “healthy” mitochondria merge into cells. Mitochondria with defects die and undergo a process of lysis or mitophagy [43,49]. Thereby, constant natural selection of mitochondria occurs and a healthy pool of mitochondria in the cell is maintained. It has been scientifically proven that the deficiency of food substrates in cells begins after 6 h of fasting. Therefore, to ensure normal mitochondrial dynamics, a person must not eat food for at least 12 h a day. For example, take a 12-h break from eating every day from 18:00 to 7:00 so that the daily fusion of mitochondria into a single mitochondrial network occurs. In the opposite case, with an excess of food substrate, mitochondria become defragmented, that is, they are in a mode of reduced bioenergetic functioning aimed at excessive production of protons. This leads to gradual degradation of the mitochondrial pool. This occurs because the cellular processes of high-quality selection of mitochondria do not work adequately due to the lack of mitochondrial need for fusion. Therefore, the cell cannot detect and destroy mitochondria with mutations and altered membrane potentials. Over time, genetic degeneration of the mitochondria increases the energy inefficiency of the cell’s functioning and, as a result, energy production at the mitochondrial level becomes increasingly inefficient [43,49,50,51,52]. Thus, an excessive number of meals (disturbed diet with constant snacking, eating late in the evening and at night) will create conditions for the occurrence of mitochondrial dysfunction as the leading pathogenetic mechanism of NCDs.

Overeating leads to an excess supply of energy substrates to the mitochondria. The task of mitochondria in this case becomes the need to dissipate more energy in the form of heat and synthesize less in the form of adenosine triphosphate (ATP) in order to reduce the metabolic oxidation state of the body. At the same time, fundamental changes in biogenesis processes occur in mitochondria with excessive generation of protons. Therefore, overeating increases oxidative stress [53]. When studying the effect of long-term overeating on the mitochondria of skeletal muscles in healthy people [54], it was found that overeating changes metabolic processes: increased fasting non-esterified fatty acid; low-density lipoprotein-cholesterol and insulin concentrations in the blood; in skeletal muscle tissue, ceramide content increased the lipid droplet content and perilipin-2 mRNA and perilipin-2 mitochondrial ribonucleic acid (mRNA) expression; and phosphorylation of AMP-activated protein kinase decreased. Overfeeding increases the expression of the mRNA of certain genes encoding mitochondrial proteins such as Citrate Synthase (CS), 2-oxoglutarate dehydrogenase complex (OGDH), Carnitine Palmitoyltransferase 1B (CPT1B), Uncoupling Protein 3 (UCP3), and Adenine nucleotide translocase-1 (ANT1). Feeding a high-fat diet has been experimentally shown to cause mitochondrial fragmentation. This leads to a decrease in the oxidative capacity of mitochondria due to a process dependent on the small GTPase Ras-related protein, Ral-A [55].

Mitochondria are membrane structures for whose adequate functioning it is important to have a complete supply of all necessary nutrients for the repair of membranes and the implementation of metabolic processes thereon. It is important to understand that all key processes depend on the morphofunctional state of mitochondrial membranes—autophagy, mitophagy, apoptosis, connection of mitochondria with the endoplasmic reticulum, and mitochondrial dynamics. The inner membrane of mitochondria plays a key role in all biosynthetic processes, since it is on it that complexes of electron transport chains are located, and it comes into contact with the mitochondrial matrix during the magnetoelectrochemical implementation of metabolism at the nanolevel of the structure of the human body. Since the membranes of cellular organelles consist of lipids and proteins, and the remodeling of these membranes is controlled by interactions between specific lipids and proteins, adequate intake of proteins and fats, and other essential nutrients into the body is vital for the adequate implementation of the processes of mitochondrial biogenesis and their normal function. The morphological state of membranes is important for the processes of normal mitochondrial dynamics and mitophagy. For example, cardiolipin is a phospholipid that promotes the formation of mitochondrial cristae and is necessary for optimal activity of respiratory complexes, assembly of supercomplexes, and optimization of the ATP synthetase enzyme. Cardiolipin deficiency leads to disruption of cristae-structure-decreased ATP synthesis, mitochondrial dynamics, mitophagy, and apoptosis. Phospholipids, in particular linoleic acid, are precursors of cardiolipin in most tissues. Accordingly, a nutritional deficiency in the supply of phospholipids and fatty acids, especially unsaturated fatty acids, can have critical consequences for the functioning of mitochondria and be a primary factor in the occurrence of NCDs [44,48,56,57,58]. Micronutrients such as coenzyme Q10 (CoQ10), zinc, copper, selenium, and iron are necessary for the efficient conversion of macronutrients into ATP [59]. Iron is necessary for oxygen transport and ATP production. Iron is the part of carrier proteins and non-heme enzymes that is involved in redox reactions and electron transfer (cytochromes and catalase) in mitochondria [60,61]. Iron is a cofactor in many key biochemical processes, including oxygen delivery and storage, mitochondrial oxidative phosphorylation, deoxyribonucleic acid replication and repair, lipid metabolism, and chromatin modification [62]. Iron is directly involved in several complexes of the mtETC, containing Fe/S clusters (complexes I, II, III and IV) and heme (complexes II, III and IV), enabling oxidative phosphorylation by ATP synthase within the mitochondria [59,63]. Selenium is a component of the amino acid selenocysteine [64], which is necessary for the formation of selenoproteins [65] such as glutathione peroxidase, thioredoxin reductase, and iodothyronine deiodinase. Glutathione peroxidases and thioredoxin reductases are essential antioxidant (redox) enzymes that are involved in preventing the harmful accumulation of intracellular hydrogen peroxide in mitochondria [66,67]. Iodothyronine deiodinases regulate the local bioactivity of thyroid hormone. This is important for stimulating mitochondrial biogenesis and increasing the mass of myocardial mitochondria, mitochondrial respiration, enzyme activity, oxidative phosphorylation, mitochondrial protein synthesis, the content of phospholipids, cytochromes, and mitochondrial DNA [68]. Methionine sulfoxide reductase B1 is also part of the human selenoproteome. Methionine sulfoxide reductase B1 controls the immune response and promotes the expression of anti-inflammatory cytokines [59]. Copper is a component of many copper-dependent proteins (known as “cuproenzymes”), including oxidoreductases, chaperones, transcriptional regulators, free radical scavengers, and immune function modulators [59,69,70,71,72]. Zinc is a critical component of the catalytic site of more than 300 enzymes. Zinc is required for the synthesis and degradation of lipids, carbohydrates, nucleic acids, and proteins [59,73]. CoQ10 plays a key role in the mtETC to facilitate ATP production. CoQ10 facilitates electron transfer from complex I (NADH coenzyme Q reductase) to complex III (cytochrome bc1 complex), and from complex II (succinate dehydrogenase) to complex III [59,60]. CoQ10 stabilizes the mitochondrial permeability transition pore and reduces apoptotic cell loss [59,74]. Therefore, poor nutrition with a chronic deficiency of nutrients important for the restoration of cell membranes, including mitochondrial ones, also gradually leads to mitochondrial dysfunction due to the occurrence of membrane morphofunctional disorders [49,75,76,77,78].

Nutrition affects mitochondria not only as an energy substrate and a source of components of their structure. It also influences the intestinal microflora, the waste products of which, together with a number of other nutrients, stimulate the biogenesis and function of mitochondria. Microbiota imbalance can be considered as a new endogenous risk factor for mitochondrial dysfunction and a promising predictor of NCDs. Dysbiosis through molecular and cellular disorders of water–salt, nutritional, and energy metabolism can lead to numerous mitochondrial dysfunctions, and the resulting oxidative stress becomes a trigger for the emergence of various epigenetic negative modifications in the expression of the genes of host cells, their microbiota and mitobiota [79,80,81,82]. This proves the feasibility of preventive introduction of prebiotic plant foods into the diet to maintain a healthy state of the intestinal microbiome in order to prevent NCDs. It is necessary to adequately supply the human body with zinc, magnesium, group vitamins (B1, B2, B3, B6, B7, B12, B5, B9), vitamin C, and vitamin E for the normal functioning of the respiratory chain [83]. At the same time, the consumption of exotoxic components with pro-oxidant activity with water, food, and air can cause both direct toxic damage and indirect damage to mitochondrial membranes due to a pathological increase in peroxide lipids and proteins [49,82,83,84,85,86]. In childhood, an exotoxic load factor can arise from the consumption of chemicalized food. In adulthood, the exotoxic load on mitochondria increases due to the consumption of alcohol, components of tobacco smoke, etc. At the whole-body level, this contributes to the progression of MD and increases the risk of developmeing CVDs and NCDs.

## 4. Physical Inactivity as an Etiological Factor of MD and NCDs

It is generally accepted that carbohydrate metabolism plays a fundamental role in the pathogenesis of NCDs. Mitochondria in skeletal muscle tissue play an important role in glucose homeostasis. They increase blood glucose clearance when insulin levels increase. All muscles, but primarily skeletal muscles due to the peculiarities of their structure, perform a bioenergetic function in the body and metabolize food energy through mitochondrial processes into other types of energy (mechanical energy, electromagnetic energy, thermal energy, acoustic energy, etc.), ATP, and glycogen. To increase bioenergetic efficiency in skeletal muscle, mitochondria are interconnected in a reticulum/network [87]. Skeletal muscle absorbs and metabolizes ~85% of all glucose [88]. Skeletal muscles make the greatest contribution to mitochondrial tissue respiration. Skeletal muscles contain the largest number of mitochondria. Therefore, their participation in the processes of mitochondrial tissue respiration is very significant for the body [89]. The amount of muscle tissue and the quantitative and qualitative content of mitochondria in its cells are fundamentally important for the existence of the human body in a state of metabolic health and adequate implementation of the phenomenon of life at the quantum level. The significance of these processes is emphasized by the fact that about 65 kg of ATP is produced and processed in the body of an adult per day. In human heart muscle, mitochondria account for about 25–30% of cell volume and consume 6 kg of ATP per day [78,90,91,92]. Therefore, physical inactivity is the second pathogenetic factor in the pathogenesis of CVDs and NCDs, since it leads to a decrease in energy expenditure in the form of mechanical movement and reduces the overall energy needs of the human body. This accordingly triggers the pathways of hypothalamic autonomic regulation and gradually leads to muscle wasting and autonomic dysfunction. Each cell (with the exception of red blood cells) contains mitochondria for its own energy supply, but striated muscle, due to the peculiarities of its structure and mitochondrial composition, produces energy for the entire body. It has been proven that physical inactivity/immobilization leads to a decrease in the number of mitochondria in striated muscles and causes mitochondrial dysfunction and an energy deficiency state in cells [49,75,93,94,95,96,97,98,99]. At the same time, excess food substrate, which has not been converted into electromagnetic, electrical, and mechanical types of energy, gradually begins to accumulate in the regional fat depots of the body, changing body composition and leading to pre-obesity, visceral obesity, and general obesity [100,101,102,103,104]. It has been proven that lack of physical activity is a pathogenetic factor for cardiovascular diseases [105,106,107,108,109,110], cancer [111,112], Alzheimer’s disease [113,114,115], type 2 diabetes [116,117], and Parkinson’s disease [118]. Low cardiorespiratory fitness is the cause of the highest percentage of all attributable fractions of all-cause mortality [119].

Physical activity is still the most optimal and proven way to improve mitochondrial function. Regular training for one month increases the content of mitochondria by 30–100%, and their volume density by 40%. Systematic regular training for six months induces systemic mitochondrial biogenesis, prevents mitochondrial DNA depletion, reduces the number of mutations, increases oxidative capacity, and improves mitochondrial morphology. Mitochondrial biogenesis increases such a parameter as the maximum absorption of oxygen by mitochondria within one minute, and also optimizes the processes of oxygen absorption for oxidative phosphorylation and oxidation of fatty acids. Muscle contraction triggers at least four intracellular signaling pathways that control mitochondrial function through mechanisms such as an increase in intracellular calcium levels, a decrease in the ATP/adenosine monophosphate (AMP) ratio, an increase in the nicotinamide-β-adenine dinucleotide/nicotinamide adenine dinucleotide phosphate (NAD+/NADH) ratio, and an increase in physiological forms as well as levels of reactive oxygen species (ROS). All these pathways, through the intermediary enzyme cyclic AMP-dependent protein kinase, affect the Phosphatidyl Glycerol-phosholipase C (PGC-1 protein), a key master regulator of mitochondrial biogenesis. ROS also act through the cell cycle regulating factor p53. However, both long-term moderate-intensity training and high-intensity interval training, as well as interval sprinting (considered a typical anaerobic exercise and taking ten minutes per week), trigger similar changes in markers of mitochondrial biogenesis: increased levels of peroxisome proliferator-activated receptor-γ coactivator-1α (PGC-1α) and prenatal glucocorticoid (GC). Although, perhaps, only long-term training causes an increase in the number of mitochondria, and short-term interval training rather affects the efficiency of existing ones, changing their ultrastructure and optimizing the functioning of the respiratory chain [120,121,122,123,124,125]. At the same time, physical inactivity causes muscle atrophy, reduces muscle strength, and forms asthenovegetative syndrome at the organismal level [95,96,97,98,99,104]. It has been proven that physical inactivity leads to a decrease in the number of mitochondria in muscles. Probably, this also involves processes of disorder in the dynamics and biogenesis of mitochondria, caused, on the one hand, by a decrease in the general energy needs of the human body, and on the other, by the general regulatory effects of the hypothalamic–pituitary–adrenal regulatory system. The human body is a complex multi-hierarchical system that has a central regulatory link to support energy and metabolic homeostasis through hypothalamic control of energy metabolism. Neurons of the hypothalamus perceive, process, and respond to signals from adipose tissue (leptin), pancreatic (insulin), and other hormonal stimuli (ghrelin, cholecystokinin, pancreatic polypeptide, etc.) by releasing neuroendocrine transmitters that stimulate or suppress hormone production. In the brain, specialized neural networks coordinate adaptive changes in food intake and expenditure. The study of mitochondrial dynamics in brain tissue has also shown that it plays a significant role in the ability of hypothalamic neurons to control glucose levels and energy homeostasis in the body. For example, in Agouti-related peptide (AgRP) neurons (hunger-promoting AgRP neurons), which stimulate appetite and regulate body weight gain, fasting leads to the separation of mitochondria, and in high-fat feeding neurons it leads to fusion. That is, the response of mitochondria is different from that of most other cells. Mitochondrial fusion in these neurons regulates electrical activity in response to a high-fat diet, stimulating the production of orexigenic peptide (AgRP-peptide), necessary for weight gain and fat deposition when nutrient excess occurs. Deletions of Mfn1 and Mfn2 in these neurons resulted in less body weight gain in rats by reducing circulating leptin levels. Pro-opiomelanocortin neurons (appetite suppressors) have the opposite function, and their mitochondrial dynamics in response to nutrient intake are different. A decrease in the expression of mitofusins in these neurons leads to disruption of the connection between mitochondria and the endoplasmic reticulum and, as a consequence, hyperphagia, leptin resistance, and obesity occur. At the same time, the amount of food eaten increases and energy consumption decreases. Thus, the body’s response to a high-fat diet depends on the patterns of mitochondrial dynamics in hypothalamic neurons. Remodeling of mitochondria in neurons ensures their response to the intake of nutrients into the body and stimulates the production of neuropeptides that either stimulate or suppress appetite, affecting metabolism at the body level. Specifically, in response to food intake, changes in body temperature, stress, or exercise, brown adipose tissue, brain, heart, or skeletal muscle adapt their metabolism to control nutrition, body weight, contractile function, antioxidant response, or insulin sensitivity [44,104,122,126]. In addition, nitroxidative stress is a major factor in the degeneration of dopaminergic neurons [127].

**Figure 1:**
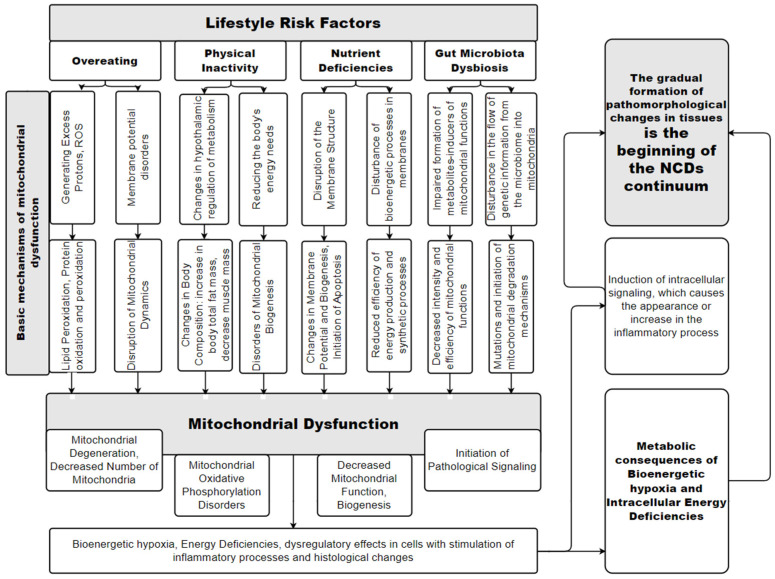
Scheme of the basic mechanisms of MD at the stage of formation of NCDs’ risk factors.

Thus, nutritional disorders and physical inactivity are basic factors that, starting from childhood, can form mitochondrial dysfunction in the cells of his body [48]. A summary of this concept is presented in Figure 1.

This scheme is an attempt to graphically display a simplified model of the mechanisms of the relationship between the basic risk factors of NCDs associated with unhealthy diet and physical inactivity, and the occurrence of NCDs through the mechanisms of MD. When analyzing the presented promising scheme of the basic mechanisms of MD at the stage of formation of NCDs’ risk factors, we can note the emergence, according to the scientific literature, of two new important risk factors for the occurrence of NCDs: nutritional deficiencies and gut microbiota dysbiosis. The participation of these risk factors, together with overeating and physical inactivity, leads to the occurrence of MD, which at this stage is manifested by mitochondrial degeneration and a decrease in the number of mitochondria, impaired oxidative phosphorylation in mitochondria, initiation of pathological signaling processes, etc. A fundamentally important point is the fact that MD leads to a pathological change in the energy state of the cell, initiating dysregulatory effects and inflammatory processes in the tissues of the human body. This becomes the basis of the “pathological circle” for the gradual occurrence of pathomorphological changes in tissues and organs and is the conditional beginning of the emergence of NCDs’ pathology, which is shown on the right side of the scheme. The term “NCDs continuum”, which is used in the scheme, will be described further in the text.

## 5. Bad Habits as an Etiological Factor of MD and NCDs

As age increases, the incidence of MD grows. According to the literature, aging is associated specifically with MD, the severity of which increases with age [128,129,130,131]. As adults, people begin to acquire bad habits. The emergence of bad habits determines additional key pathogenetic mechanisms of the beginning and progression of the direct damaging effect of external chemical agents on mitochondria. The pathological effects on mitochondria of tobacco smoking [132,133,134,135], alcohol consumption [136,137,138], consumption of chemicalized food products [139,140,141,142], drug use and irrational drug therapy [143,144,145,146,147,148,149,150,151], and chemical pollution of the human environment have been proven [152,153,154]. In this case, the specificity of the manifestations of MD will be determined by the individual characteristics of the tissues that have undergone toxic effects and the type of mitochondrial damage in these tissues.

From the perspective of systems medicine, the following mitochondrial “targets” of exotoxic effects have been identified:

- *Electron transport chain.* The toxic effect leads to a decrease in its functions, primarily to disorders of tissue respiration processes and the occurrence of bioenergetic tissue hypoxia in cells, when, despite a sufficient amount of oxygen and food substrate, disorders occur with their absorption and ATP synthesis. At the tissue–organism level, energy deficiency and decreased resistance occur [44,48,154,155,156,157].
- *Electron transport disorders in complexes I, II, and III of the mitochondrial electron transport chain*. Pathological generation of superoxide radical occurs, followed by the formation in reactions with other molecules of the mitochondrial matrix of hydrogen peroxide, peroxynitrite, hydroxyl radical, lipid hydroperoxide, and lipid radicals. Conditions are created for self-sustaining processes of lipid peroxidation and oxidative damage to proteins and nucleic acid cells. At the tissue–organism level, inhibition of cell function, accumulation of RNA and DNA mutations, and degenerative changes in tissues and organs occur [44,48,154,155,156,157].
- *Oxidative Phosphorylation*. Toxic damage leads to inhibition of lipid β-oxidation, which causes disorders of lipid homeostasis of cells with the accumulation of potentially toxic lipid products and pathological activation of lipid peroxidation [48,158,159,160].
- *Membrane components of mitochondria and mitochondrial functional proteins*. The consequences of violating their integrity and functioning may be different. Mobilization of cytochrome C into the intermembrane space activates caspases and the process of apoptosis. The release of endonuclease G and AIF initiates cell death through a caspase-independent mechanism. The release of thanatogenic proteins increases the number of membrane pores and initiates a scenario of cell death [161,162,163].
- *Mitochondrial nucleic acids*. The appearance and accumulation of mutations can lead to depletion of the mitochondrial DNA pool, changes in synthesis processes, disorders of mitochondrial biogenesis with an increase in low-quality mitochondria, and a decrease in their total number [164,165,166].

It is clinically important that these mechanisms of mitochondrial disorders are interrelated. They can occur simultaneously, in parallel and/or sequentially, form processes like a “vicious circle” and, as a result, cause the appearance of a complex of degenerative changes in mitochondria. The gradual progression of mitochondrial dysfunction leads at a quantum level to changes in the membrane biopotential of cells, changes in signaling functions between cells, and the appearance of dysregulatory effects, both in cells and in the tissues of the organ they form. In this case, overproduction of ROS can induce intracellular signaling mechanisms that cause the appearance and/or increase in the activity of the inflammatory process and the occurrence of morphological changes in tissues (hypertrophy, fibrosis, atherosclerosis, etc.). The final mitochondrial consequences of toxic effects are disorders of the bioenergetic state of cells, the occurrence of cytoenergy deficiency, damage to mitochondrial DNA with the subsequent initiation of integrative processes of mitochondrial dysfunction in the form of activation of the mechanisms of mitochondrial degradation [48,167].

**Figure 2:**
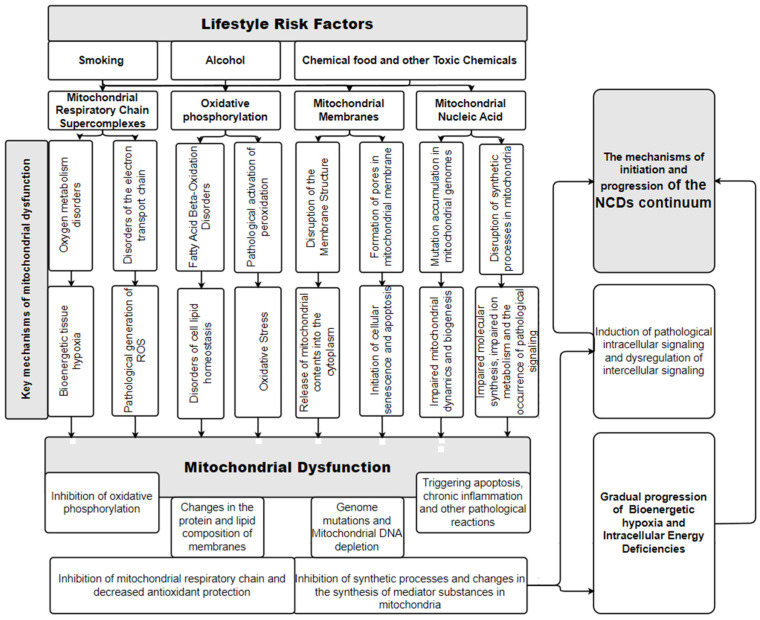
Scheme of the key mechanisms of MD at the stage of formation of NCDs risk factors.

Thus, toxic exposures are key factors that have multiple direct damaging effects on mitochondria and cause MD. This leads to progression of NCDs [47]. A summary of this concept is presented in Figure 2.

This scheme is an attempt to graphically display a simplified model of the mechanisms of the relationship between the key risk factors of NCDs associated with toxic effects on the human body and the occurrence of NCDs through the mechanisms of MD. These NCDs’ risk factors have a direct damaging effect on mitochondria and disrupt their functions. The resulting mitochondrial dysfunction is associated with serious biochemical disturbances in the metabolic functioning of cells. Intracellular energy deficiency and the appearance of pathological cellular signaling become the pathogenetic basis for pathological histomorphological changes in cells and tissues of organs, and this is the basis for the progression of NCDs.

It is important to note that the presented schemes (Figure 1 and Figure 2) complement each other and are universal simplified models of the relationship between NCDs’ risk factors and the pathogenesis of NCDs through MD for all tissues of the human body. Of course, in vivo, the degree and features of the manifestation of MD will be different in the tissues of different organs.

## 6. The Role of MD in the Occurrence and Progression of NCDs

The pathological functioning of mitochondria in the cells of an organ (Figure 1 and Figure 2) leads to the fact that the tissues of this organ begin to have impaired metabolism. This will clinically manifest itself in dysfunction of this organ and the appearance of corresponding symptoms of the disease. Therefore, the pathogenetic significance of the stage of formation of risk factors lies in the gradual occurrence of metabolic, immune, and morphological changes in organ tissues, which will then lead to the manifestation of NCDs.

It is fundamentally important to note that it is the occurrence of mitochondrial dysfunction that leads to pathological changes in cell metabolism in NCDs. It happens because mitochondria perform key functions of energy supply to the cell and intercellular communication. It is mitochondria that ensure the adequacy of the cytoenergetic state of tissues, organs, and the human body by performing the following key functions: energy supply, synthesis, and signaling [93,168].

Mitochondria provide energy synthesis in the form of ATP on electron transport chains in the tricarboxylic acid cycle and oxidative phosphorylation [92,168]. Mitochondria take an active part in biosynthetic processes associated with polypeptide, amino acid and fat catabolism, the formation and metabolism of urea, organic acids, in the biosynthesis of heme, nucleotides, steroids, cardiolipin, ubiquinone, various metabolites, and signaling molecules. Mitochondria function as a platform for the generation of simple (containing one carbon atom) and complex (containing two or four carbon atoms) carbon-containing products based on fatty acids, pyruvate, acetate, ketoglutarate, and many amino acids capable of causing a variety of biological effects, both locally and systemically [51,52,81,93,127,161,168,169,170,171,172]. Mitochondria are a physiological source of reactive oxygen species and free radicals—anion radical (O^2−^), hydroxyl radical (^–^OH), non-radical oxidants (hydrogen peroxide (H_2_O_2_), singlet oxygen (^1^O_2_), nitric oxide (NO), peroxynitrite, lipid hydroperoxides, alkoxyl radical, peroxyl radical ^–^OOH, sulfate radical ^–^SO_4_, etc.). Under physiological conditions, the generation and destruction of ROS in mitochondria is controlled by enzymatic and non-enzymatic reactions and is an integral part of the physiological regulation of metabolic processes [46,127,168,170]. Mitochondria play an important role in maintaining the buffer capacity of ions (primarily calcium ions) in the cytoplasm of cells, which are necessary for the operation of many internal signaling pathways, primarily neuronal synapses [46,78,83,84,85,86,168,170,171,172]. Mitochondria are also a source of signaling molecules in healthy cells. There is a constant two-way signaling information interaction between mitochondria and the nuclear genome, in which nuclear genes regulate the biogenesis of mitochondria in cells, their quantitative content, functional activity, and the processes of auto- and mitophagy. At the same time, mitochondria are active participants in the metabolic reprogramming of both individual and all cells of the human body [49,50,75,78,82,86,168,173,174,175,176]. Mitochondria are involved in the body’s immune response. For example, various components of mitochondria (mitochondrial DNA, N-formyl-methionine proteins, polypeptides, cytochrome C, ATP, cardiolipin, ROS, etc.) can activate dendritic cells and macrophages. Mitochondrial DNA and other mitochondrial components in the extracellular space and body fluids can induce local and/or systemic inflammatory processes [86,177,178,179]. Mitochondria are able to initiate the formation of inflamasomes and activate caspase-1, which facilitates the secretion of pro-inflammatory cytokines, interleukin-1, -18, and other inflammatory mediators [46]. Mitochondrial DNA and ROS released by eosinophils are key components of the innate immune response and antimicrobial defense [52,86,93,177,178,179,180,181,182,183,184]. In case of serious damage, when a significant amount of mitochondria is released, induction of the activation of monocytes, neutrophils, and inflamasomes, which take part in inflammatory processes, is noted [76,78,185,186]. Mitochondria are involved in intracellular signaling, which controls cell proliferation and differentiation, thereby maintaining tissue homeostasis. The participation of mitochondria in the processes of aptotosis, aging mechanisms, and other forms of cell death has been proven [76,78,85,127,167,180,183,185,187]. Mitochondria control the level of sex hormones and take part in steroidogenesis [48]. It is also categorically important that biologically active low-molecular compounds formed by representatives of the symbiotic microbiota and mitochondria can interact with similar cellular receptors of different tissues. Bacterial and mitochondrial DNA can be incorporated into the host nuclear genome. Excessive generation of ROS by mitochondria can interfere with the structure of intestinal microbiocenosis and damage the integrity of the epithelial barrier of the gastrointestinal tract. It is currently believed that a significant part of the universal pathogenetic mechanisms of the diseases of internal organs, in particular overproduction of free radicals and systemic chronic inflammation, are the result of the general effects of mitochondria and microbiota [45,177,188,189].

Mitobiota and microbiota are considered as the source of most endogenous enzymes, substrates, cofactors, and regulators involved in the epigenetic processes of mitochondria, cellular chromatin, symbiotic, and pathogenic microorganisms [48,49,75,90,190,191,192]. It has been established that the regulation of mitochondrial functions in different tissues occurs not only due to the expression of nuclear genes of the mitobiota but is also supported by the additional entry of genetic information into the mitochondria from the microgenome present on the human skin and mucous membranes. Mitochondria and symbiotic microbiota jointly participate in energy synthesis and epigenetic modification of the microbial, nuclear, and mitochondrial genome through DNA methylation, chromatin modeling, and microRNA expression. It has been proven that the ability of microbiota to form such key microbial metabolites as short-chain fatty acids, lactate, urolithins, hydrogen sulfide, etc. is important for the functioning of mitochondria. Thus, the qualitative and quantitative composition of the intestinal microbiota influences mitochondrial functions [79,80,177,192,193,194].

A very serious conclusion emerges that mitochondria are involved in a significant number of epigenetic processes in the human body, and they have a key energetic and metabolic significance for the existence of the cell and ensuring the possibility of realizing the phenomenon of life. There is also a significant amount of scientific research on the pathogenetic role of mitochondrial dysfunction in almost all acquired diseases of the internal organs of the gastrointestinal [195,196,197,198,199,200,201,202,203,204,205], respiratory system [174,184,206,207,208], urinary system [209,210,211,212,213,214,215], cardiovascular system [78,216,217,218,219], oncological diseases [220,221,222,223,224], endocrine disorders [77,78,187,225,226,227,228,229], neurodegenerative diseases [76,85,127,170,171,172], in pediatrics [30,48,230,231,232,233] etc. This indicates the systematic implementation of the mechanisms of occurrence of comorbidity in NCDs due to mitochondrial dysfunction.

That is why the resulting MD leads to disorders of tissue respiration, tissue bioenergetic hypoxia, cytoenergy deficiency, and changes in the membrane potential of cells with the formation of disregulatory effects in tissues. This gives rise to the metabolic consequences of cellular cytoenergy deficiency, bioenergetic hypoxia, and induces pathological intracellular signaling, which may cause an increase in the activity of inflammatory processes, the triggering of apoptosis, and other mechanisms of cellular aging and death. This ultimately triggers the process of pathological changes in tissues, which is the beginning of the NCDs continuum. Therefore, another important conclusion is that MD is a proven universal pathogenetic mechanism that organically complements the existing theory of the cardiovascular continuum and explains the quantum mechanisms of metabolic pathogenesis at the microlevel of the structural organization of the human body. However, how is mitochondrial dysfunction related to the phasing of visceral organ involvement in NCDs?

## 7. Is There a Valid NCDs Continuum?

**Figure 3:**
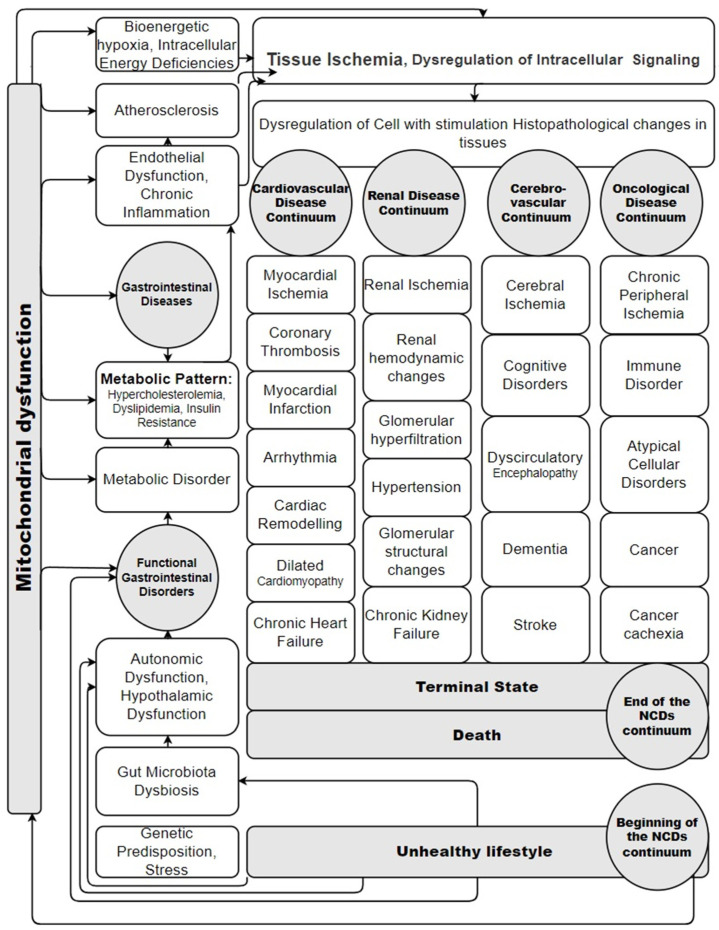
The promising scheme for the NCDs continuum has been developed.

Are there stages of involvement of organs and organ systems in the pathogenesis of NCDs? Is there a pattern and logic in the sequence of occurrence of pathology in NCDs? The correct answer to these questions will be obtained when all the pathogenetic mechanisms of NCDs’ progression are studied and disclosed. But the idea that NCDs are a chain of sequentially occurring pathological conditions and diseases has already been put forward for consideration [48,234]. The theoretical pathogenetic basis of this idea is that mitochondrial dysfunction occurs gradually and not in all organ tissues at once. Mitochondrial functions are selectively disrupted, apparently first in the cells of the digestive tract and liver [201,205]. It happens because they bear the greatest burden of detoxifying chemical components and metabolizing excess food during overeating. Therefore, it is logical that the mitochondria of the digestive tract organs will be the first to disrupt their functions under the influence of NCDs’ risk factors. Then, MD of hepatocytes will lead to disruption of the synthetic function of the liver and to the appearance of pathological signaling [203]. The consequence of this will be the gradual emergence of a metabolic pattern with dyslipidemia and conditions for the occurrence of chronic inflammation and atherosclerosis [235,236]. Over time, MD will occur in more and more organs and tissues, and it will cause a disruption in the energy and metabolic processes in them and a disruption in their function. Long-term, multi-year gradual progression of mitochondrial dysfunction ultimately leads to the appearance of the characteristic comorbid pathology of NCDs. Thus, it is the gradually increasing MD that causes the emergence and progression of systemic pathological damage to organ tissues in NCDs. And this causes the gradual development of comorbidity in NCDs [234]. Therefore, it would be logical and correct to consider NCDs in a continuum model: they are a series of pathological conditions that are combined, characterized by gradual progression and the emergence of a natural sequence of disease manifestations, which gradually change and complement existing previous pathological disorders over time. However, the overall NCDs continuum includes the cardiovascular continuum as an integral part of one of its stages (Figure 3).

The prototype of this scheme is the Cardiovascular Disease Continuum scheme, which was developed by V. Dzau and E. Braunwald [22,23,24,25,26,27,28]. The scheme presented in the review is a working model and an attempt to describe the systemic relationship between the development of all groups of NCDs in the whole organism, taking into account the role of MD. It is logical that NCDs’ risk factors (unhealthy lifestyle) are at the beginning of the continuum and lead to the occurrence of MD. As practical experience shows, functional gastrointestinal disorders and gastrointestinal diseases create the metabolic basis for the development of many pathogenetic mechanisms of NCDs (endothelial dysfunction, chronic inflammation, atherosclerosis, etc.). This leads to specific pathological changes in the tissues of the corresponding organs. Exactly how this happens and why different combinations of NCDs occur in different patients continues to be studied. This is an important and not fully understood issue for modern medicine. But it is certainly obvious from the literature that mitochondrial dysfunction leads to these pathological continuums in different tissues, and this depends on the influence of NCDs’ risk factors.

Perhaps the concept of a continuum of NCDs is an important promising approach for further search for solutions to the problem of NCDs. Expanding the medical perspective from the cardiovascular continuum theory to the NCDs continuum theory may allow the creation of a model of the gradual development and phasing of comorbidity progression. This can also help further study the systematicity and complexity of the interaction of organs in the human body during diseases. The theory of the cardiovascular continuum is generally accepted today. It is the theoretical basis on which the understanding of the development processes of the most important CVDs is based [23,24,25,26,27,28,237,238,239,240]. The cardiovascular continuum is a continuous chain of interconnected changes in the cardiovascular system, starting from exposure to risk factors and the gradual onset and progression of CVDs to the development of terminal cardiac damage with a fatal outcome. Until now, the cardiovascular continuum has been considered separately from other NCDs in science. A number of authors have attempted to represent the pathology of other organs and systems in terms of a continuum, for example, the renal continuum in parallel with the cardiovascular continuum [25,239], the continuum between primary low-renin arterial hypertension and primary hyperaldosteronism [238], and the cardiometabolic continuum [241].

From a clinical perspective, the concept of a continuum of NCDs has merit. It can serve as a model for describing the occurrence of comorbidity with the gradual development of pathology of other organs and systems during their developmental progression. Now, comorbidity is one of the central problems of internal medicine. As practical experience demonstrates, patients with pathological abnormalities in only one organ (i.e., monomorbid) are young people and isolated cases in the practice of internal medicine doctors. The number of organs that are involved in the pathological processes of NCDs increases with the age of the patient. At the same time, the severity of the pathology and the number of NCDs increases [237]. Therefore, multimorbidity and comorbidity have long been recognized as fundamental signs of a modern patient in the 21st century. Over the past decades, the views of the entire medical community have been focused on studying the issues of comorbidity.

The features of the course and combination of various NCDs with each other have been studied, but the holistic picture and mechanisms of the emergence and progression of comorbidity have not yet been completely reduced to a unified system of knowledge; they continue to remain fragmented and require a generalization of a systemic medical approach. It is important for the clinician to know where the patient is on the cardiovascular and NCDs continuum, as this will determine the appropriateness and scope of preventive interventions for the patient. That is why a scholastic approach to the patient with an assessment of all his comorbid pathology is important from a practical point of view for determining and understanding the stage of the general continuum of NCDs (and the cardiovascular continuum as its component) for the formation of correct, adequate, individual preventive treatment tactics in accordance with requirements of P4 medicine in the management of patients with NCDs. P4 medicine is based on the strategies, technologies, and analytical tools of systems medicine. P4 medicine makes it possible to provide predictive, preventive, and personalized care to the patient and includes a ‘participatory’ component.

Of course, the issue of the mutual influence of comorbid pathological conditions on the part of organs and systems in NCDs remains insufficiently studied and continues to be studied. Therefore, the fact that MD in various forms and degrees of expressiveness is a universal mechanism that can explain what is happening in the tissues of various organs in NCDs is absolutely scientifically important.

## 8. Prevention and Diagnosis of Mitochondrial Dysfunction

The mechanisms of the occurrence of MD (Figure 1 and Figure 2) categorically substantiate the logic of the formation of measures to prevent the occurrence of NCDs. Therefore, popularizing a healthy lifestyle among all segments of the population in order to prevent NCDs is extremely important. Of course, measures to prevent MD and NCDs should be aimed at combating the entry into the human body of toxic substances (alcohol, tobacco smoke, etc.) and chemical agents unnatural for the body (microplastics, etc.). Therefore, it is necessary to continue the fight against bad habits (tobacco smoking and use of drugs, including alcohol), and it is advisable to organize human life in an environmentally friendly environment. A promising therapeutic and prophylactic aspect may be the use of drugs that have a positive effect on mitochondrial function. It is important to understand that risk factors for NCDs need to be diagnosed as early as possible and that all people, from childhood onwards, be actively encouraged to: prevent excessive intake of food substrates (overeating); avoid frequent, constant meals (it is advisable to eat a diet of 3–4 times a day with a nightly fast interval of at least 12 h; short-term fasting); ensure adequate nutritional nutrition; avoid physical inactivity and exercise daily; and ensure the consumption of ecological, natural food products with sufficient fiber content and normal shelf life (products without chemical preservatives).

Diagnosis of MD is difficult. Mitochondrial functions can be assessed using methods such as:

- measurement of the corresponding oxidative enzymes involved in oxidative phosphorylation (citrate synthase and succinate dehydrogenase) [11,242,243,244];
- technology for measuring mitochondrial respiration and substrate utilization in skeletal muscle using the Oroboros technology method, which provides modular high-resolution respirometry systems for studying mitochondria and cells and allows the measurement of respiration at controlled oxygen levels in combination with redox biology (nicotinamide adenine dinucleotide (NADH) and CoQ10), reactive oxygen species production, mitochondrial membrane potential, ATP production, and Ca^2+^ or pH [11,242,245];
- technology for measuring mitochondrial respiration and substrate utilization in skeletal muscle using the Seahorse technology method, which makes it possible to simultaneously measure mitochondrial respiration and glycolysis in living cells in real time [11,245,246];
- a method for assessing mitochondrial respiration based on nuclear magnetic resonance (NMR) technique [11,247,248,249];
- a method for assessing mitochondrial respiration based on the magnetic resonance spectroscopy (MRS) technique [11,250,251].

The problem is that simple and convenient methods for clinical diagnosis in practical medicine have not yet been developed. Existing methods for assessing mitochondrial functions are focused on uses for research purposes. Their fundamental disadvantages are high cost, technical complexity, and in some cases, invasiveness or the need to grow a cell culture.

Considering the fundamental importance of diagnosing MD for practical healthcare, the development of methods for direct or indirect assessment of mitochondrial functions that will be suitable for widespread use in clinical practice is an urgent scientific task. The scientific search for suitable methods continues. For example, according to the authors [252], the technique they developed for indirectly measuring mitochondrial function and metabolic flexibility can be implemented on a large scale and on an outpatient basis. The method is based on a combination of measuring fat oxidation by indirect calorimetry using stoichiometric equations and measuring blood lactate levels during exercise, which are important mitochondrial substrates. During exercise, both fats and lactate are oxidized in mitochondria, as they are important mitochondrial substrates. Decreased fat oxidation capacity and increased blood lactate during exercise may indicate decreased mitochondrial function [11,252,253,254].

In our opinion, another promising method for indirectly assessing metabolic processes at the tissue level can be techniques for analyzing the emission of biophotons. Ultra weak photon emission is a universal biological phenomenon of radiation in the visible range, which is a manifestation of the phenomenon of life [255,256,257,258,259]. The emission of biophotons appears to be directly related to mitochondrial function and is most likely the result of their metabolic activity and signaling [260]. Registration of this phenomenon can also be carried out in different ways and is also technologically complex. However, in a number of studies we used the technology of the electrophoton emission analysis method using a simple mobile certified measuring device [261,262,263,264]. This made it possible to record significantly different metabolic activities in tissues non-invasively in vivo in patients with NCDs and functionally healthy people [263,264]. This is preliminary encouraging data, but for final conclusions it is necessary to continue research in this direction.

But how can physicians assess mitochondrial function in a clinical setting until adequate objective assessment techniques are developed? MD is a very significant diagnostic parameter, and, knowing the important role of mitochondria in the progression of NCDs, it cannot be ignored.

The largest number of mitochondria are localized in striated muscle cells. The more developed the striated muscles are, the correspondingly more adequately functioning mitochondria are in the body. This is why athletes have higher metabolic parameters and better health. Based on this idea, a simple accessible indirect method for assessing mitochondrial function can be a bioimpedance study of body composition with an objective determination of the percentage of muscle content in the human body according to its age norm [265,266,267,268]. A study conducted to assess the muscle content in a patient’s body showed that, in patients with NCDs, muscle content is lower than the age norm and the emission of biophotons is reduced [104]. At the same time, in the history of modern medicine, there are recorded medical cases where long-term regular training against the background of professional correction of the diet in people of the older age group led to a significant development of muscle mass and a decrease in the manifestations of NCDs. The most obvious example of this is the personal case of the American Therapist Dr. Jeffry Life [269], who changed his lifestyle and achieved good personal results in solving his own NCD problem. This technique absolutely deserves detailed study and close attention of the scientific world in terms of its effect on mitochondrial function. This experience may become the basis for the creation of unified medical protocols for the prevention and treatment of mitochondrial dysfunction in the future.

## 9. Discussion and Future Prospective

According to the literature, mitochondrial dysfunction is a pressing problem that scientists have been actively studying over the past few decades. Of course, modern science now has a large amount of new important data on the functioning of mitochondria in various diseases of the human body. However, these new data are very slowly and limitedly integrated into the practical medical industry. Evidence of this is the fact that practicing doctors do not yet use mitochondria as a “therapeutic target” in the process of treating patients. Official guidelines for correcting mitochondrial dysfunction have not yet been developed. Knowledge about mitochondrial dysfunction as one of the key mechanisms of the occurrence and progression of NCDs is just beginning to be implemented in medical education and more widely popularized among doctors of various specialties. However, there are a number of obstacles in the way of these processes. One of these obstacles is the lack of a generally accepted theoretical concept of views on the role of mitochondrial dysfunction in the occurrence and progression of NCDs. According to the literature, a number of scientists are successfully working in this direction [9,10,11,270,271].

This review has contributed a certain amount to the development of these ideas. Based on a systematic analysis of scientific data, a theoretical concept of modern views on the occurrence of mitochondrial dysfunction under the influence of risk factors for NCDs is presented. Also, the idea of the existence of an NCDs continuum was presented to the attention and discussion of scientists. Many issues require continued scientific discussion and further research. But it should be noted that a new look at NCD risk factors through the prism of the role of mitochondrial dysfunction may justify completely different approaches to their prevention and treatment in the future. Understanding the fundamental mechanisms of the role of mitochondrial dysfunction provides a fresh perspective on the harms of an unhealthy diet and the benefits of regular exercise. This may help address obesity and other metabolic problems that contribute to the onset and progression of NCDs. Therefore, now the focus of scientific interest on mitochondrial dysfunction is increasingly shifting from mitochondrial diseases to all NCDs [270,271]. However, as noted earlier, aspects of the occurrence of comorbid pathology continue to be studied. It remains unclear why and how certain combined pathologies of organs arise in NCDs. How is mitochondrial dysfunction, which occurs in the cells of some internal organs, related to metabolic processes in other internal organs?

The lack of methods for routine, simple diagnosis of mitochondrial dysfunction that would be convenient for use in clinical medicine is the second obstacle to the applied use of knowledge about mitochondrial dysfunction. However, existing scientific trends in this matter give hope that in the future this problem will also be solved. Today, methods for objectively assessing the content of muscle tissue in the body can still be used in clinical practice to indirectly assess the metabolic state of patients [265,266,267,268].

Pharmacological studies of substances that have the ability to positively influence mitochondrial function continue. The importance of using nutritional supplements that improve mitochondrial function (for example, carnitine, CoQ10, creatine, vitamin B2, etc.) in the complex treatment of patients with NCDs is becoming increasingly scientifically obvious and proven [270,271].

Innovative approaches are exploring the use of mitochondrial transplantation as an advanced and promising treatment for mitochondrial dysfunction. Experiments with using healthy mitochondria to replenish or replace damaged mitochondria have shown promise in preclinical trials of various diseases [271].

Promising issues for further scientific research remain the need to study the cause-and-effect relationships between MD in the tissues of various organs, which lead to the development of comorbidity in NCDs. The scientific direction of searching for pharmacological agents to correct MD and its metabolic consequences is also relevant.

## 10. Conclusions

(1) Mitochondria are an important therapeutic target. (2) MD can begin in childhood under the influence of basic factors of an unhealthy lifestyle (overeating with poor diet, nutritional deficiencies, physical inactivity, toxic load on the body). (3) MD can occur and progress in adulthood under the influence of key factors of an unhealthy lifestyle that have a direct damaging effect on mitochondria (smoking, alcohol, and other toxic substances). (4) The development of MD in NCDs can be described in a continuum model, which begins in childhood under the influence of unhealthy lifestyle factors (malnutrition and physical inactivity) leading to functional disorders of the digestive organs, the occurrence of MD in the cells of their tissues, the emergence of a metabolic pattern, and conditions for the occurrence of atherosclerosis. Further progression of mitochondrial dysfunction leads to metabolic disorders in the cell, pathological signaling, and the occurrence of pathological histomorphological changes. Thus, MD is a universal mechanism that causes organ dysfunction and comorbidity. Continued progression of MD aggravates pathological changes in cells and leads to complications and the end of the continuum—to the death of the organism. (5) Prevention of MD involves diagnosing and eliminating the factors that cause it: poor diet, physical inactivity, and exposure to toxic agents on the human body (tobacco smoke, alcohol, and other chemicals). (6) MD is an important diagnostic parameter, the assessment of which at this stage is only possible for research purposes.

The search for methods for assessing mitochondrial functions that can be used in widespread medical practice continues. In this case, in a clinical setting, it is possible to objectively determine the percentage of muscle mass in the human body using the method of bioimpendansometry. A decrease in the percentage of muscle in the body below the age norm is an objective parameter that indicates a decrease in the number of mitochondria and mitochondrial dysfunction.

## Data Availability

Not applicable.
